# Extra Virgin Olive Oil Polyphenols: Modulation of Cellular Pathways Related to Oxidant Species and Inflammation in Aging

**DOI:** 10.3390/cells9020478

**Published:** 2020-02-19

**Authors:** Gabriele Serreli, Monica Deiana

**Affiliations:** Department of Biomedical Sciences, University of Cagliari, 09042 Monserrato, Cagliari, Italy; gabrieleserreli@hotmail.it

**Keywords:** extra virgin olive oil, aging, polyphenols, NF-κB, antioxidants, anti-inflammatory activity, hydroxytyrosol, tyrosol, oleuropein

## Abstract

The olive-oil-centered Mediterranean diet has been associated with extended life expectancy and a reduction in the risk of age-related degenerative diseases. Extra virgin olive oil (EVOO) itself has been proposed to promote a “successful aging”, being able to virtually modulate all the features of the aging process, because of its great monounsaturated fatty acids content and its minor bioactive compounds, the polyphenols above all. Polyphenols are mostly antioxidant and anti-inflammatory compounds, able to modulate abnormal cellular signaling induced by pro-inflammatory stimuli and oxidative stress, as that related to NF-E2-related factor 2 (Nrf-2) and nuclear factor kappa-light-chain-enhancer of activated B cells (NF-κB), which have been identified as important modulators of age-related disorders and aging itself. This review summarizes existing literature about the interaction between EVOO polyphenols and NF-κB and Nrf-2 signaling pathways. Reported studies show the ability of EVOO phenolics, mainly hydroxytyrosol and tyrosol, to activate Nrf-2 signaling, inducing a cellular defense response and to prevent NF-κB activation, thus suppressing the induction of a pro-inflammatory phenotype. Literature data, although not exhaustive, indicate as a whole that EVOO polyphenols may significantly help to modulate the aging process, so tightly connected to oxidative stress and chronic inflammation.

## 1. Introduction

For over a decade, several studies have been showing that adherence to an olive oil-centered Mediterranean diet is associated with lower mortality and extended longevity [1,2]. The wholesome properties of extra virgin olive oil (EVOO) have been extensively investigated and, as recently discussed among experts from the International Olive Council [3], strong evidence suggests a preventive role against the most common age-related degenerative diseases as cardiovascular and neurodegenerative disorders, as well as cancer and diabetes [3]. Thus, consuming EVOO as part of a balanced diet improves prognosis and promotes a “successful aging”, affecting health-span and, as a consequence, lifespan. However, it has been recently shown in cellular, animal, and human models, as summarized in the excellent review by Fernandez del Rio et al. [4], that EVOO is also able to virtually modulate all the features of the aging process, the so called “hallmarks of aging”. Aging is now recognized as a multifactorial process involving diverse and complex alterations, deemed exactly as the "hallmarks of aging", as genomic instability, mitochondrial impairment, stem cell exhaustion, loss of proteostasis, epigenetic alterations, telomere attrition, deregulated nutrient sensing, reshaped intercellular communications, and cellular senescence [5]. EVOO consumption has been shown to attenuate most of the aging-related alterations due to the presence of high monounsaturated fatty acids (MUFA) and, particularly, of its minor bioactive compounds. EVOO may induce epigenetic changes, modulate proteostasis and nutrient sensing alterations, and seems to affect telomere length through the enhancement of telomerase activity [4]. However, the healthful effects of EVOO consumption in aging seem to be mostly related to the renowned anti-inflammatory and antioxidant activities of its phenolic fraction. If it is actually true that the aging process is multifactorial, it is also indisputable that inflammation and oxidative stress are some of the most consistent outcomes of increasing age in cells and tissues and, whether such factors are causes or consequences of aging, they are considered a common thread throughout most of the hallmarks of the process [6]. The polyphenols contained in EVOO have been demonstrated, for instance, to contribute to the maintenance of genomic stability, thanks to the capacity to protect DNA (nuclear and mitochondrial) against oxidative stress-induced harm [7,8] and to inhibit mitochondrial dysfunction sustaining endogenous antioxidant defenses (both non-enzymatic and enzymatic), thus attenuating the aging-related raise of lipid peroxidation [4]. They are also able to delay cellular senescence and alteration of intercellular communication pathways [9], likely through the modulation of the chronic inflammation (an example is steatohepatitis) that is strictly tangled with the aging process [10,11]. Most of the intracellular pathways switched on in response to inflammatory and oxidative stresses, recently identified in humans as important modulators of aging and age-related diseases, are those related to nuclear factor kappa-light-chain-enhancer of activated B cells (NF-κB) and NF-E2-related factor 2 (Nrf-2) [12]. The chronic activation of NF-κB signaling is a common feature of numerous age-related and inflammatory diseases, but it has also been associated with aging itself. NF-κB hyper-activation has been shown to directly induce cellular senescence [13,14,15] and associated secretory phenotype [16], as well as to enhance the level of pro-inflammatory mediators. Nrf-2 level has been shown to decrease with age, as a result of epigenetic suppression or enhanced expression of its negative regulators [17], leaving tissues more vulnerable to oxidative stress and thus triggering accelerated aging, contributing to each of the hallmarks of the process [18]. The purpose of the present review was to summarize the outcomes of several in vivo and in vitro studies, which recently revealed the interaction of biologically relevant EVOO polyphenols and their metabolites with the major NF-κB and Nrf-2 related cellular pathways, strengthening the opinion that such polyphenols may exert beneficial effects on aging. Scopus and Pubmed databases were searched for articles in this context and the criteria for article selection was based on the novelty and relevance of the papers, particularly selecting those providing possible mechanisms underlying effects on the abovementioned signaling pathways. A total number of about 250 papers were examined for this review, searching for keywords like “NF-κB”, “Nrf-2”, “aging”, and “olive oil polyphenols”. Moreover, the cross references of the selected papers were also taken into consideration through Scopus search.

## 2. Absorption, Metabolism, and Bioavailability of EVOO Phenolic Compounds

**Figure 1:**
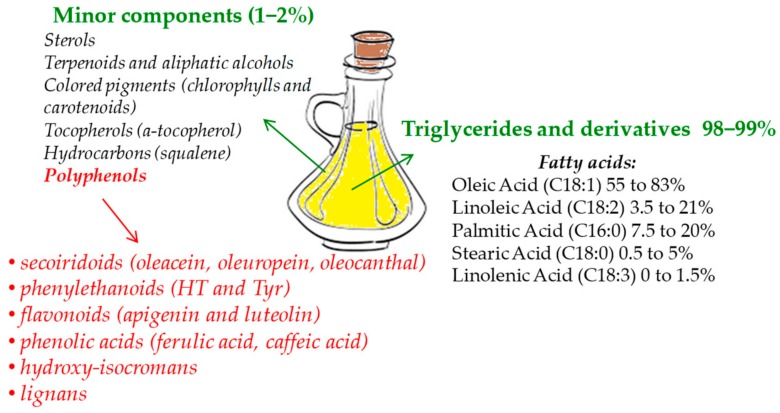
Extra-virgin olive oil (EVOO) main components.

Polyphenols compose the hydrophilic fraction of EVOO, which is only a small portion with respect to lipophilic compounds [19,20] (Figure 1). The phenolic fraction consists of a few tens of compounds, although in reality, not all of them are found together in every EVOO [21]. All these compounds pertain to different chemical subclasses, are present in a broad range of concentrations [20], and basically belong to six subclasses: Secoiridoids (dialdehydic forms of decarboxymethyl elenolic acid linked to tyrosol (Tyr) or hydroxytyrosol (HT), oleacein, oleuropein, and oleocanthal) [22,23], phenylethanoids (HT and Tyr) [22], flavonoids (apigenin and luteolin) [23], phenolic acids (for instance, ferulic acid, caffeic acid, and gallic acid) [23], hydroxy-isocromans (1-(39-methoxy-49-hydroxy)phenyl-6,7-dihydroxy-isochroman and 1-phenyl-6,7-dihydroxyiso -chroman) [24], and lignans ((+)-1-acetoxypinoresinol and (+)-1-pinoresinol) [25]. Most of the studies concerning the bioactivity of these polyphenols aimed to show the numerous properties of two phenols, HT and Tyr, which are the most concentrated in EVOO together with elenolic esters oleocanthal, oleuropein-, and ligstroside-aglycons [20,26].

Once ingested with the diet, an absorption of 40%–95% of HT and Tyr occurs in humans [27]. Moreover, it is well known that they might be absorbed and display their biological activity in a dose-dependent manner [28]. It was also demonstrated that the large majority of these compounds are found in human urine and plasma, though as conjugated forms such as glucuronides, sulfates, and methylates [26,29]. Once in the stomach environment, EVOO polyphenols can be moderately modified: Aglycone secoiridoids such as oleuropein and oleocanthal usually undergo a time-dependent hydrolysis in the stomach, causing a considerable raise of free Tyr and HT amounts after 30 min. This hydrolyzation of aglycone secoiridoids is proportional to gastric residency, even though under physiological conditions some of them do not go through hydrolyzation but enter the small intestine as such [30]. Vice versa, if the ingested secoiridoids are glycosilated, they cannot be subjected to gastric hydrolysis [27] and therefore, unmodified glucosides of oleuropein, as well as relevant concentrations of free Tyr and HT, may be absorbed by the small intestine. Following their absorption in the small intestine, the levels of Tyr and HT increase quickly, reaching a peak concentration at different time frames for human plasma (1 h) and urine (2 h) [29]. Vissers et al. [27] indicated that, after EVOO polyphenols intake, their absorption strictly depends on the different polarities of the diverse phenolics structures. Manna et al. [31] studied in Caco-2 cell monolayers the mechanisms of intestinal HT absorption and showed that HT transport takes place via a bidirectional passive diffusion mechanism. Furthermore, it has also been demonstrated that, after a relevant absorption of EVOO phenolics at gastrointestinal level, bioavailability of these compounds is conversely low, due to an intensive metabolization at various levels [20,29,32]. 

Indeed, while crossing enterocytes, HT and Tyr as well as other EVOO phenolic compounds undergo substantial metabolism involving phase II transformations. The predominant metabolites of Tyr and HT, namely glucuronides, sulfate, and methylates, are formed by the respective action of glucuronosyltransferases (UDPGT), sulfotransferases (SULT), and catechol-*O*-methyl transferases (COMT) [20,33]. Moreover, acids and aldehydes coming from oxidation of the aliphatic alcohols [34], as well as acetylated and *N*-acetylcysteine derivatives, [35] can be found. After HT and Tyr intake, *O*-glucuronidated conjugates were found as the most concentrated metabolites in human plasma and urine [36,37], while studies on HT bioavailability in rat urines demonstrated that glucuronide and sulfate metabolites are by far the most copious among the HT phase II metabolites [35]. In addition, Rubio et al. [38] detected other metabolites in human plasma, namely homovanillic acid, homovanillic acid sulfate, and HT acetate sulfate. Kountouri et al. [39] instead found relative high concentrations of 3,4-dihydroxyphenylacetic acid and homovanillyl alcohol in human urines, in addition to the aforementioned metabolites. Equally relevant is in fact the metabolic pathway of HT involving the COMT, which gives rise in vivo to the biosynthesis of homovanillyl alcohol [40].

Once absorbed, HT and Tyr together with their metabolites are widely distributed in the entire organism [20]. In rats fed with HT, in relevant nutritional amounts, it was demonstrated that HT and its phase II metabolites (HT glucuronide and sulfate) as well as homovanillyl alcohol could be stored dose-dependently in the kidney, brain, and liver [41]. Previously, it has been shown that HT [42] and Tyr [43], as well as HT sulfate and HT acetate sulfate [42], may cross the blood brain barrier and go through brain uptake in rats. Moreover, still in rats, it was shown an extensive and rapid uptake of these compounds by several organs including heart, lungs, and skeletal muscle [34]. 

## 3. Modulation of Nrf-2 and Antioxidants Enzymes by EVOO Polyphenols

Aging is not only the consequence of the accumulation of oxidative stress-dependent harm [44], as stated by the “free radical theory of aging” developed in the 1950s [45]; even so, the oxidative damage resulting from redox and antioxidative capacity imbalance, primarily caused by the Nrf-2 age-related decline, is still recognized to play a pivotal role [46]. The transcription factor Nrf-2 mediates the general adaptive response of the cell, managing proteostasis, metabolism, and inflammation [47,48,49], but its predominant function is to be the principal regulator of oxidative protection, being a key protein in the transcriptional expression of several antioxidant-metabolizing enzymes [50,51]. Nrf-2 belongs to the NF-E2 family of nuclear basic leucine zipper transcriptional activators [52,53], which is largely bound to Kelch-like ECH-associated protein 1 (Keap1) and is retained in the cytoplasm under normal physiological conditions. After dismantling of the Nrf-2-Keap1 complex by inducers, Nrf-2 undergoes prompt translocation into the nucleus where it triggers its target genes in heterodimeric combinations with other transcription factors [54]. Following translocation into the nucleus, Nrf-2 binds to antioxidant response elements (ARE) in the promoter regions of its target genes and induces the expression of phase II detoxification enzymes and antioxidant proteins, such as superoxide dismutase (SOD), c-glutamylcysteine synthetase (c-GCS), the rate-limiting enzyme in the glutathione (GSH) synthesis pathway, glutathione S-transferase (GST), cystine/glutamate exchange transporter, glutathione peroxidase (GPx), thioredoxin reductase (TRX), heme oxygenase-1 (HO-1), and NADPH quinone oxidoreductase-1 (NQO1) [50,51]. In addition, Nrf-2 recently showed healthful effects against apoptosis caused by mitochondrial toxins and Fas signaling [51]. 

Multiple studies have shown a significant interaction between Nrf-2 (and related antioxidant enzymes) expression and intake of EVOO phenolics with the diet (Table 1). For instance, senescence-accelerated mouse-prone 8 received diets with 10% olive oil characterized by either high (HP) or low amounts of olive oil polyphenols (LP) for 4.5 months. Nrf-2, as well as its target genes paraoxonase-2 (PON2), c-GCS, NQO1, and GST were then measured in the hearts of these aged mice. It was seen that mRNA levels of antioxidant genes were remarkably elevated in heart tissue of the HP as compared to the LP group. This result was related to the level of HT present in the HP oil through additional mechanistic cell culture experiments, which showed a direct involvement on the induction of Nrf-2-dependent gene expression [55]. Still regarding HT, its supplementation in high-fat diet (HFD)-fed male mice C57BL/6J (daily portions of 5 mg/kg) mitigated the metabolic alterations produced by HFD, keeping the efficacy of Nrf-2 at normal levels, reducing the fall of the peroxisome proliferator-activated receptor-α (PPAR-α) activity and attenuating NF-κB activation [56]. In the same model, HT (10–50 mg/kg/day) was also capable to efficiently elevate liver and muscle GST activity, which was reduced by HFD [57]. In addition, eight-week administration of 60 mg/kg/day of oleuropein considerably lowered pro-inflammatory cytokines and blood pressure and increased the levels of Nrf-2 dependent phase II enzymes, such as HO-1 and NQO-1, in spontaneously hypertensive rats (SHR) in comparison with the saline-treated SHR rats [58]. In mice treated with lipopolysaccharide (LPS) to induce acute lung damage, improvement of HO-1 expression related to Nrf-2 activation was instead observed in mice treated orally with Tyr (240 mg/kg) [59]. At the hepatic level, EVOO polyphenols are thought to be effective in enhancing Nrf-2 activation and the consequent antioxidant enzymes release [10]. An interesting study led by Barrera et al. [60] showed instead that enriched EVOO (100 mg/day) administration in male Wistar rats did not modify neither Nrf-2 activation nor its upstream signaling, whereas it was found to be able to limit the significant increase of Nrf-2 and antioxidant enzymes levels provoked by an iron-rich diet (200 mg iron/kg diet). Still, in the liver, the same amount of EVOO in a model of high-fat diet (HFD) in mice was also effective in inducing a normalization of oxidative stress related parameters, with mechanisms that did not involve Nrf-2 modulation [61]. 

With regards to GPx and other enzymes, there are conflicting data in literature reporting its modulation by EVOO phenolics, probably depending on the tissue localization of the enzyme. For instance, 60-day-old Wistar male rats received 7.5 mg/kg of HT daily for 30 days, which did not improve GPx and GSH concentration, but rather increased oxidative stress in heart tissue, perhaps due to its too high concentration [62]. Conversely, in male Wistar rats where TCDD (2, 3, 7, 8-tetrachlorodibenzo-*p*-dioxin)-induced hepatotoxicity led to a reduction in the activity of catalase (CAT) and GPx, treatment with olive oil, Tyr and HT along with TCDD inhibited the oxidative damage by reinstating GPx and CAT levels. Moreover, TCDD treatment showed to reduce HO-1 and NQO1 activities in rat liver, which were restored by olive oil, HT, and Tyr treatment [63]. Still in rats, exposure to a toxic agent, 2,4-D (2,4-dichlorophenoxyacetic), led to liver injury and oxidative damage followed by the considerable decrease of GPx, CAT, SOD, and glutathione reductase (GR) enzymes levels, compared to controls. Vice versa, treatment with olive oil or hydrophilic extract in combination with 2,4-D enhanced the levels of antioxidant enzymes, revealing activities comparable to those of the untreated rats [64]. A similar outcome occurred in mice, where CAT and GPx activities in the islet of Langerhans were 25% higher in olive-oil-treated mice than in those untreated and higher than in those treated with sunflower oil [65]. In elderly humans (aged 65–96 years), a significant increase of CAT in erythrocytes and a decline in GPx and SOD activity were instead observed after EVOO with high oleuropein derivative intake [66]. 

Regarding the in vitro tests, which were performed in the last 10 years, oleuropein, HT, and Tyr, as well as more complex EVOO phenolic extracts, were evaluated in a broad variety of cell sorts. They resulted in being able to enhance Nrf-2 expression and consequently HO-1, γ-glutamyl-cysteinyl-ligase (γ-GCL), NQO1, GPx, TRX, SOD, and other antioxidant enzymes in LPS-treated macrophages [67,68], but also in different cancer cells [69,70,71,72,73] or normal cells, like retinal pigmented epithelium ARPE-19 cells [74], untreated or stimulated with pro-oxidant and pro-inflammatory stimuli.

## 4. Modulatory Function of EVOO Polyphenols on NF-κB Expression Pathway and Related Inflammatory Response

Age-related reduction of Nrf-2 is coupled to an increase of NF-κB activity. Activation of these two factors occurs in response to similar stimuli but implies a complex and dynamic interplay that, based on cell features and tissue context, aims at counterbalancing each other activity [75]. As age progresses, the decrease of Nrf-2 activity is not able to compensate for the activity of the NF-κB system, thus contributing to the aging-related hyperactivation of NF-κB signaling [76]. NF-κB is the transcription factor most involved in the aging process [77]. Its activation is associated with diverse upstream signaling pathways related to immune attacks, growth factors, and internal and external danger signals [78,79]. It regulates expression of hundreds of genes [80] that are mainly implicated in inflammatory responses and immune signaling, as those encoding cytokines and chemokines (tumor necrosis factor alfa—TNF-α and interleukins), growth factors and their receptors, pro-inflammatory enzymes (inducible nitric oxide synthase—iNOS and cyclooxygenase-2—COX-2 above all), other transcription factors, but also genes that regulate apoptosis and cell-cycle progression [81,82]. The increase of NF-κB pathway activity is associated with a whole-body persistent, low-level inflammation that characterizes the aging process [83,84]. This phenomenon is called “inflammaging” and, besides accompanying aging, it plays a key role in the onset and progression of age-related degenerative diseases, which, in addition to disease-specific etiological factors, have been associated with low-grade inflammation triggered and supported by oxidative stress [2]. An inappropriate NF-ĸB signaling pathway activation characterizes, in fact, numerous age-associated disorders including neurodegeneration, cardiovascular diseases, osteoporosis, obesity, type 2 diabetes, sarcopenia, and cancer [78,85,86].

EVOO phenolics have widely been recognized as anti-inflammatory agents also for their capacity to modulate improper activation of NF-ĸB signaling, thus limiting its deleterious effects in all tissues. Actually, their ability to modulate NF-ĸB is tightly linked to their antioxidant nature. It is well known that EVOO polyphenols, besides enhancing enzymatic endogenous antioxidant defenses, possess direct free radical scavenging and radical chain breaking capacity. Moreover, the catecholic structure of some of them (namely oleuropein, HT and derivatives, for example) may also prevent reactive species formation via metal chelation features. They may counteract the formation of a broad range of ROS (O_2_^−^ above all) and RNS, like the peroxynitrite anion (ONOO^−^) [87,88,89]. Beyond direct oxidant effects, ROS play an essential role in intracellular signaling pathways modulation and activity of manifold transcription factors, including NF-κB and activator protein 1 (AP-1) and hypoxia-inducible factor-1α (HIF-1α) [90]. Thus, modifying cellular redox status, EVOO phenolics may control the activation of those signaling pathways, such as NF-κB, directly implicated in the cellular response to pro-inflammatory and oxidative stimuli. One of the most studied upstream constituents of the NF-κB signaling pathway is the activation of the mitogen-activated protein kinases (MAPKs) [91]. It is in fact well known that oxidants have the capacity to activate the extracellular signal regulated kinase (ERK) pathway. Several studies have indicated that ROS-mediated ERK activation occurs through modulation at the level of growth factor receptors, p21Ras, and Src kinases. Moreover, protein 38 (p38), c-Jun *N*-terminal kinase (JNK), and the mitogenic ERK were shown to be noticeable targets of ROS [90]. Another target of ROS release that is involved in the upstream activation of NF-κB is the serine/threonine kinase Akt (also known as protein kinase B or PKB). It is a pivotal oncogenic effector of phosphoinositide 3-kinase (PI3K) signaling and positively modulates ROS/RNS production through direct involvement in the activation of NADPH oxidase and mitochondrial bioenergetics. In addition, the PI3K cascade activated through Akt modulation can in turn be potentiated by cellular ROS increasing levels [92]. Excessive oxidative stress is thought to activate PI3K/Akt signaling by repressing the functionality of its negative regulator phosphatase and tensin homolog (PTEN), one of the most commonly altered tumor suppressor genes in cancer. In addition, PI3K/Akt signaling can be also modulated by ROS through the regulation of the protein tyrosine phosphatases (PTPs), which inhibit the receptor tyrosine kinases (RTKs) such as PDGFR and EGFR through dephosphorylation [92]. The Akt-mediated ROS production is often associated with increasing of cellular senescence [93,94] and correlates with accelerated tumorigenesis. 

EVOO polyphenols modulating action on NF-ĸB was observed both in vitro and in vivo in different tissues and cell lines (Table 2). In vivo, HT effectively modulated caspase-3 and NF-ĸB p65 subunit levels, attenuating apoptosis in rat brain cells [95], while in high-fat diet (HFD)-fed mice, daily doses of HT (5 mg/kg) down-regulated NF-ĸB together with PPAR-α in the liver [58]. In female BALB/c mice, after induction of systemic lupus erythematosus (SLE)-like disease, feeding with a saleable EVOO picual variety, containing high amounts of phenolics (600 ppm), exerted a protective role in the management of such autoimmune disease, likely through the inhibition of MAPK, JAK/STAT, and NF-κB pathways in splenocytes [96]. The consumption of a VOO highly concentrated in phenol (398 ppm of phenols like HT, Tyr, and minor components) based breakfast was also tested in humans, where it significantly limited the activation of NF-ĸB postprandial gene expression in peripheral blood mononuclear cells, in comparison with the consumption of VOO with a low or intermediate polyphenol content [97]. 

In vitro, EVOO extracts or simple phenols demonstrated, particularly in monocytes and activated macrophages, the ability to inhibit MAPKs activation, which in turn caused inhibition of phosphorylated-IκBα and thus NF-κB expression and translocation into the nucleus. These effects have been noticed in particular in those cells treated with oleuropein aglycone [105], Tyr [98,106], and HT [104], as well as complex phenolic extracts [107,108]. At the intestinal level, the breakdown of the NF-κB inhibitor IĸBα was counteracted by EVOO phenolic extracts or by simple phenols HT and Tyr glucuronide and sulfates in intestinal Caco-2 cells, where NF-κB was experimentally activated by a mixture of dietary oxysterols [99] or by LPS [88]. Pretreatment of colon cancer HT-29 cells with HT was also successful in the decrease of NF-ĸB expression by modulating Akt/PKB and ERK 1/2, which was induced by TNF-α [103]. HT also suppressed angiogenesis, tumor growth, and induced apoptosis and cell cycle arrest by suppressing the activation of NF-κB and Akt pathways both in human hepatocellular carcinoma cells (HCC) in vitro and in an orthotopic model of human HCC in vivo [102]. In mouse mammary epithelial cells (MMECs), HT attenuated NF-κB activation induced by Staphylococcus aureus, by down-regulating MAPK p38, JNK, and ERK1/2 activation [101]. Lastly, to study the effects of EVOO phenols in managing inflammatory autoimmune diseases, Rosillo et al. [100] tested HT and HT acetate, as well as an EVOO phenolic extract, in synovial fibroblasts SW982 cells treated with interleukin (IL)-1β. It was observed that IL-1β-induced MAPKs phosphorylation, the secretion of pro-inflammatory mediators, and the matrix metalloproteases activity were significantly decreased together with the NF-κB activation. 

### 4.1. Inhibition of COX-2 and Prostaglandins

COX-2 is the inducible form of cyclooxygenase, which is usually expressed at the site of tissue damage to release prostaglandins. Indeed, this enzyme converts arachidonic acid to prostaglandin G2 and prostaglandin H2, a precursor for the biosynthesis of prostaglandin D2, E2 (PGE2), F2α and I2, and thromboxane A2, led by specific synthases [109,110]. The NF-κB signaling system plays a pivotal role in COX-2 expression, that occurs at low levels in normal cells and is activated in response to chemical, biological, physical, or UV light stimuli [111]. COX-2 is also up-regulated after stimulation led by growth factors, inflammatory cytokines, and tumor promoters. COX-2 is associated with aging, which may accelerate pathogenic processes such as cancer [112]. It has been verified to be over-expressed in approximately 80% of adenocarcinomas and in 40% of human colorectal adenomas in comparison to normal epithelial cells [113]. Increased COX-2 expression has also been suggested to be involved in the development and/or progression of other age-related diseases including atherosclerosis, diabetes, osteoporosis, and Alzheimer’s disease [114,115,116,117]. Being one of the key mediators of the inflammatory process, it has been directly related to the aging process itself, although the mechanism of its action in aging tissues, in relation to different pathological conditions, is still not clear [118,119].

As anti-inflammatory and anticancer agents, EVOO polyphenols were supposed to be also effective in combating COX-2 over-expression (Table 3). In fact, EVOO phenolic extracts (50 µg/mL) showed inhibitory effects on undifferentiated intestinal Caco-2 cancer cells proliferation, CREB (cAMP response element-binding protein), and p38 phosphorylation, resulting in a downstream lowering in COX-2 expression [120]. Moreover, the same research group found that HT alone (5.0–200 µM) exerted strong antiproliferative effects against Caco-2 cancer cells through its capacity to induce a block of cell cycle in G2/M. However, HT effects seemed to be linked to a downstream decrease of cyclin D1 expression and a strong inhibition of ERK1/2 phosphorylation, rather than the inhibition of COX-2 expression and p38 phosphorylation [121]. In another intestinal cancer cell line, namely HT-29, oleocanthal and p-HPEA-EDA (decarboxymethyl ligstroside aglycon) repressed cell viability and induced apoptosis via AMPK (5′ adenosine monophosphate-activated protein kinase) activation and COX-2 suppression [122]. Cusimano et al. [123] showed that oleocanthal effects were even more effective than the traditional commercially accessible COX inhibitors (nimesulide, indomethacin, ibuprofen) in suppressing cell growth in colorectal carcinoma (SW480, HT-29) and hepatocellular carcinoma (Huh7, Hep3B, HepG2, and PLC/PRF/5) models. 

Oleuropein showed an inhibitory effect against COX-2 over-expression in different tumor cell lines [130], but the most studied EVOO phenolic compound is undoubtedly its derivative HT. It was shown, for instance, that LPS-induced expression of COX-2 and thus PGE2 production were inhibited by HT pretreatment in human and mouse granulocytes, macrophages, and monocytes [66,100,124,125,126,127]. Among the mechanisms involved in COX-2 suppression by HT, as well as by EVOO polyphenols extracts, the inhibition of NF-κB following MAPKs modulation is the most recognized [107]. In vivo, it was observed that HT suppressed COX-2-induced inflammation and PGE2 production in a carrageenan-induced rat paw edema model, although the efficacy was less than that of celecoxib and indomethacin [124]. The same outcome was observed in diet-dextran sodium sulfate (DSS) mice which developed chronic colitis, where olive oil supplemented with HT or with phenolic extracts (850 ppm), given to enrich normal diet, resulted to be more efficacious than olive oil in down regulating cytokines release like TNF-α, MAPKs, and NF-κB pathway activation and consequently iNOS and COX-2 expression [125,126]. Still in mice, BALB/c type were administered with Tyr (0.1–10 mg/kg orally) 1 hour prior to an intratracheal injection of LPS (25 μg/50 μL) to induce acute lung injury. Treatment with Tyr reduced pro-inflammatory cytokines IL-1β, interleukin (IL)-6, and TNF-α, as well as iNOS, COX-2, and phosphorylated-IκBα in lung tissue and bronchoalveolar lavage fluid [98].

### 4.2. iNOS Modulation

iNOS is an enzyme mostly transcriptionally regulated and it is not normally produced in most of the cells [131]. It is usually induced by cytokines, mainly expressed in macrophages, neutrophils, and epithelial cells [132] and it has been shown to be involved in several human illnesses associated with inflammation. At intestinal level, for example, under pathological conditions, an enhancement of iNOS expression leads to an over production of nitric oxide (NO). This condition determines a loss of barrier function with epithelial disruption and bacterial translocation, which triggers the inflammation [133,134]. NO has in fact been recognized as involved in the etiology of this kind of mucosal inflammation such as Crohn’s disease and ulcerative colitis [135]. An upregulated production of NO also provokes harmful effects through the generation of RNS, such as ONOO^−^, dioxide nitrogen (NO_2_), and the nitroxyl anion (NO^−^), all accountable for oxidative stress [136]. Hence, elevated levels of ONOO^−^ formed after the reaction of large quantities of NO with the superoxide anion, may be an essential factor in tissue injury [137]. Thus, a significant number of researches have been carried out in the last decade, with the purpose of assessing EVOO phenols efficacy in contrasting NO overproduction related to iNOS expression (Table 3). Recently, our research group showed, in Caco-2 differentiated as normal enterocytes, that glucuronide and sulfate metabolites of HT and Tyr, together with the free forms, were capable to counteract the LPS-induced release of NO. All the tested metabolites suppressed NO release, functioning as inhibitors of iNOS expression following inhibition of NF-κB activation through IĸBα phosphorylation and modulation of MAPKs [88]. In the same cell model, we also observed that EVOO phenolic extracts were able to limit oxysterols-mediated NO and cytokines overproduction, by modulating MAPK-NF-κB pathway and consequently iNOS expression [99]. 

Generation of NO by iNOS is also implicated in critical functions such as microbial killing and immune regulation [138]. Under homeostatic conditions, NO takes part in a host-beneficial system to kill pathogens when local infection occurs, but its release can also be a critical factor in the propagation of tissue injury in conditions such as abdominal malignancies, sepsis, and autoimmune disorders [139]. Extensive research highlighted the importance of EVOO polyphenols in limiting NO production in immune system cells, treated with LPS to simulate inflammatory conditions. HT above all [67,104,140] but also oleuropein [67,108] and complex EVOO polyphenols extracts [107,127] were able to impede NO overproduction in monocytes and macrophages. These effects occurred actually not only in these cell types, but also in almost all the tissues studied, both in vitro [129,141] and in vivo [98,142].

Although most of the papers did not investigate the mechanism involved in NO and iNOS down regulation, what emerges is the capacity of EVOO polyphenols to modulate the signaling cascade, which leads to NF-κB activation, starting from Akt and MAPK ERK1/2, p38, and JNK1/2/3 and subsequently involving IKK/IĸB. Indeed, the result of limiting phosphorylation of NF-κB inhibitor IĸB is reflected in a blockage of NF-κB translocation into the nucleus and therefore of COX-2 and iNOS expression. 

## 5. Conclusions

EVOO polyphenols are known to be principally antioxidants in the broadest sense of the word, being able to directly scavenge oxidant species and to increase cellular endogenous antioxidant defenses. In that way, they may control cellular redox balance and consequently several redox-sensitive signaling pathways also related to inflammation as NF-κB and Nrf-2 pathways. These two transcription factors that control each other have been recognized to also be a crucial participant in the oxidative stress and inflammatory responses related to both aging and age-related disorders. Several studies indicate that EVOO polyphenols, mainly HT, Tyr, and oleuropein, can activate Nrf-2 signaling and dependent genes expression, inducing a cellular defense response against oxidative injuries and pro-inflammatory stimuli. Activating Nrf-2, EVOO polyphenols also suppress NF-κB-dependent inflammatory response, but they are also involved in the direct inhibition of NF-κB activation, through the modulation of upstream kinases, the inhibition of its inhibitor IĸB degradation or the blockage of NF-κB nuclear translocation. HT and Tyr are the most active modulators of NF-κB signaling. It is noteworthy that some of their phase II metabolites, sulphates, and glucuronides, showed the ability to retain such efficacy, since they are more concentrated than parent compounds, if not the only ones present, in most target tissues. Oleocanthal has been shown to suppress COX-2, a key enzyme of the inflammatory response, with an efficacy comparable to that of the classical commercially available inhibitors; as a result of NF-κB modulation, most of EVOO polyphenols also control the activity of iNOS.

**Figure 2:**
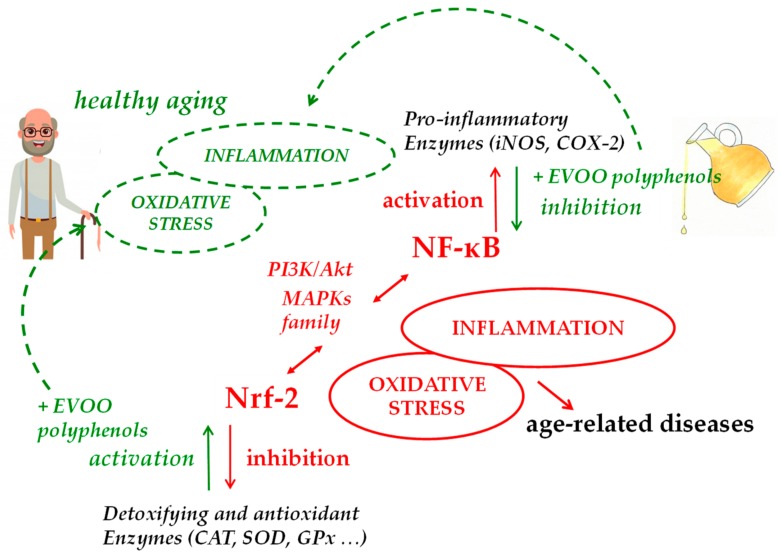
Main molecular pathways involved in EVOO polyphenols health effects in aging.

Data collected so far on the ability of EVOO polyphenols and their metabolites to modulate cellular pathways related to ROS and inflammation have shown significant effects in animal models and in vitro, supporting the growing in vivo evidence of their beneficial effects on aging. Although further investigation is needed to elucidate possible mechanisms underlying these effects on the complex process of aging, the repression of NF-κB and the activation of Nrf-2 signaling are likely to be the key mechanisms of the antioxidant/anti-inflammatory action of EVOO polyphenols, which may impede the appearance of a pro-inflammatory phenotype in several age-related disorders and during the aging process itself (Figure 2). Thus, EVOO polyphenols-rich dietary supplements, present in a wide variety of products on today’s market, or much better, the regular consumption of EVOO as the principal dietary fat within a balanced Mediterranean-type diet, can potentially confer additional benefits that help slow aging, improving health and lifespan.

## Figures and Tables

**Table 1:** EVOO polyphenols and modulation of Nrf-2 and antioxidant enzymes.

Compound or Diet Supplement Tested	In Vivo/In Vitro Model	Concentration Tested	Effects	References
	**In vivo**			
Diet with 10% olive oil with high (HP) or low (LP) phenol content	Senescence-accelerated mouse-prone 8	Pelletized Western-type diet with 20% fat, in which 10% of fat was from olive oil HP (532 mg gallic acid/kg oil) or LP (44 mg gallic acid/kg oil)	mRNA levels of antioxidant genes were significantly increased in heart tissue of the HP with respect to the LP group	[55]
	High-fat diet (HFD)-fed male mice C57BL/6J	5 mg/kg daily	Maintenance of the activity of Nrf-2 at normal levels, reduction of the drop of PPAR-α activity and attenuation of the NF-κB activation	[56]
HT	High-fat diet (HFD)-fed male mice C57BL/6J	10–50 mg/kg daily	Elevation of liver and muscle GST activity	[57]
	60-days old Wistar male rats	7.5 mg/kg/day for 30 days	Inefficacy to enhance GPx and GSH concentration. Increasing of oxidative stress in heart tissue, perhaps due to too high concentrations	[62]
	Male Wistar rats with TCDD (2,3,7,8-tetrachlorodibenzo-*p*-dioxin)-induced hepatotoxicity	0.5 mg/kg, oral	Restoration of the level of CAT, GPx, HO-1 and NQO1 activities in rat liver	[63]
Oleuropein	Spontaneously hypertensive rats (SHR)	Eight-weeks administration of 60 mg/kg daily	Increase of the levels of Nrf-2 dependent phase II enzymes NQO-1 and HO-1	[58]
Tyr	Mouse model of acute lung injury	240 mg/kg	Improvement of (HO)-1 expression related to Nrf-2 activation	[59]
Olive oil	Male Wistar rats with TCDD (2,3,7,8-tetrachlorodibenzo-*p*-dioxin)-induced hepatotoxicity	10 mL/kg, oral	Restoration of the level of NQO1, CAT, HO-1 and GPx activities in rat liver	[63]
Olive oil or its hydrophilic fraction	Male adult Wistar rats	300 µL/ day	Increased the levels of CAT, GPx, GR and SOD enzymes	[64]
EVOO	12 to 16-wk-old male OF1 mice	50 µL/day	Increased the levels of GPx and CAT	[65]
	Elderly humans (aged 65–96 years)	50 mL/day	Significant raise of CAT was found in erythrocytes and, conversely, a decrease in GPx and SOD levels	[66]
	**In vitro**			
	Macrophages RAW264.7 stimulated with LPS	10 μM	Induction of Nrf-2 nuclear translocation	[67]
	Macrophages J774 A.1-mediated oxidation of LDL	0.5 mM	Preservation of GPx and GR mRNA expression	[68]
	Human hepatocarcinoma HepG2 cells	0.5, 1, 5 and 10 µM	Increase of the activity and the expression of GPx, GR and GST. Increase of Nrf-2 expression	[69]
	Human hepatocarcinoma HepG2 cells	10–40 µM	Keeping of GSH concentration and increase of GPx	[70]
HT	IMR-32 human neuroblastoma cells	5 µM	Upregulation of Nrf-2 expression	[71]
	Caco-2 cells treated with acrylamide	5–40 µM	Keeping of GSH concentration and increase of GPx	[72]
	PC12 pheochromocytoma cells treated with 6-OHDA	0–50 μM	Increase of the expression of HO-1, GCL, NQO1 and thioredoxin reductase following activation of the Keap1-Nrf-2 pathway	[73]
	Retinal pigmented epithelium ARPE-19 cells	100 μM	Preservation of Nrf-2 levels and antioxidant enzymes HO-1, NQO-1	[74]
Tyr	Macrophages J774 A.1-mediated LDL oxidation	0.5 mM	Preservation of GPx and GR mRNA expression	[68]

**Table 2:** Modulation of Akt, MAPK, and NF-κB pathway by EVOO polyphenols.

Compound or Diet Supplement Tested	In Vivo/In Vitro Model	Concentration Tested	Effects	References
	**In vivo**			
HT	Rat brain from adult male Sprague-Dawley rats	6 weeks administration 100 mg/kg/ daily	Modulation of NF-κB p65 subunit and caspase-3 levels	[95]
	High-fat diet (HFD)-fed mice	5 mg/kg	NF-κB and PPAR-α down-regulation in the liver	[56]
EVOO	Female BALB/c mice with systemic lupus erythematosus (SLE)-like disease	10% of oil in a complex diet	Inhibition of MAPK, JAK/STAT and NF-κB pathways in splenocytes	[96]
VOO	Peripheral blood mononuclear cells of humans(19 men, 30 women)	40 mL during breakfast	Limitation of NF-κB postprandial gene expression	[97]
Tyr	BALB/c mice orally administered with LPS	0.1–10 mg/kg	Inhibition of phosphorylated-IκBα and NF-κB traslocation into the nucleus	[98]
	**In vitro**			
Tyr glucuronide Tyr sulfate HT glucuronide HT sulfate	Caco-2 cells	1 µM	Inhibition of IĸBα degradation and of p38 and ERK1/2 activation. No effects on Akt phosphorylation	[88]
EVOO phenolic extracts	Caco-2 cells	5–25 μg/mL	. Inhibition of p38 and ERK1/2 activation and of IĸBα degradation	[99]
	Human sinovial SW982 cells	12.5–50 µM	Inhibition of MAPKs phosphorylation and NF-κB activation	[100]
	Mouse mammary epithelial cells stimulated with *Staphylococcus aureus*	10–40 μg/mL	Attenuation of NF-κB activation by downregulating MAPK p38, JNK and ERK1/2 activation	[101]
HT	Human hepatocarcinoma HepG2 cells	0–400 µM	Inhibition of NF-κB and PI3/AKT pathways activation	[102]
	HT-29 intestinal cells	200 μM	Decrease of NF-ĸB expression by modulating ERK 1/2 and Akt/PKB	[103]
	Macrophages J774 A.1 stimulated with LPS	50–200 μM	Prevention of NF-κB traslocation into the nucleus	[104]
Oleuropein	Monocyte-like THP-1 cells	0–50 μM	Decrease of NF-ĸB expression	[105]

**Table 3:** Inhibition of cyclooxygenase-2 (COX-2) and inducible nitric oxide synthase (iNOS) over-expression by EVOO polyphenols.

Compound or Diet Supplement Tested	In Vivo/In Vitro Model	Concentration Tested	Effects	References
	**In vivo**			
Tyr	BALB/c mice orally administered with LPS	0.1–10 mg/kg	Suppression of iNOS, COX-2 expression and phosphorylated- IκBα	[98]
HT	Carrageenan-induced rat paw edema model	500 mg/kg	Selective COX-2 inhibition	[124]
EVOO phenolic extract enriched with HT	Diet-dextran sodium sulfate (DSS) mice	40 mg/kg	COX-2 suppression after inhibition of NF-κB and MAPK pathways	[125,126]
	**In vitro**			
Tyr glucuronide Tyr sulfate HT glucuronide HT sulfate	Caco-2 cells	1 µM	Inhibition of LPS-induced NO over-release and iNOS expression. Inhibition of p38 and ERK1/2 activation and of IĸBα degradation	[88]
	Caco-2 cells	5–25 μg/mL	Inhibition of LPS-induced NO over-release and iNOS expression. Inhibition of p38 and ERK1/2 activation and of IĸBα degradation	[99]
EVOO phenolic extracts	Macrophages J774 A.1 stimulated with LPS	50–150 μg/mL	Inhibition of NO over-production and of COX-2 and iNOS expression	[127]
	Peritoneal macrophage of mice stimulated with LPS	25–50 μg/mL	Induction of a significant downregulation of iNOS and COX-2 through reduced nuclear translocation of NF-kB following MAPK phosphorylation	[107]
	Undifferentiated Caco-2 cells	50 μg/mL	Inhibition of CREB and p38 phosphorylation and consequent reduction in COX-2 expression	[120]
HT	Macrophages RAW264.7 stimulated with LPS	10 μM	Inhibition of NO over-release and of iNOS expression	[67]
	Macrophages J774 A.1 stimulated with LPS	50–200 μM	Down-regulation of COX-2 and iNOS gene expression by preventing NF-κB translocation into the nucleus	[104]
	Undifferentiated Caco-2 cells	5–200 µM	Inhibition of ERK1/2 phosphorylation. No effects on p38 activity and COX-2 expression	[121]
	LPS-activated peripheral blood mononuclear cells	100 µM	Reduction of COX-2 gene expression and PGE2 secretion	[128]
Oleuropein	Macrophages RAW264.7 stimulated with LPS	10 μM	Inhibition of PGE2 and NO over-production. Reduction of iNOS and COX-2 expression	[67]
	LPS-activated human primary osteoarthritis chondrocytes	1–5 μM	Inhibition of NO over-production following inhibition of iNOS expression through MAPK modulation	[129]
Oleocanthal	Hepatocellular carcinoma (Huh7, PLC/PRF/5, HepG2 and Hep3B) and colorectal carcinoma (HT29, SW480) models	25 μM	Inhibition of COX-2 expression	[123]
	HT-29 intestinal cells	1–5 μg/mL	Induction of apoptosis through COX-2 suppression and AMPK activation	[122]
